# Medical Schools

**Published:** 1849-01

**Authors:** 


### THE WESTERN JOURNAL.

THIRD SERIES.—VOL. III.—NO. I.

LOUISVILLE, JANUARY 1, 1848.

### MEDICAL SCHOOLS.

No candid and impartial observer, we believe, will deny that there is a tendency in our country to an excessive multiplication of medical schools. Rapid as is the increase of our population, the rate of increase in these institutions is greater. For a long time, the only schools of medicine in America were at Philadelphia and New York. The first medical degrees conferred were in 1770, by the latter school, and the graduating classes of both schools, for two years, numbered only thirteen. In 1804 Dr. John B. Davidge commenced a course of lectures in Baltimore to a class of six students, and three years subsequently a charter was granted by the State for the University of Maryland. The medical department of Yale College, and the Boston medical school were organized in 1810. In 1818, medical schools were commenced at Lexington and Cincinnati, and in 1824 the medical college of South Carolina was established.

Thus we see, that it is only thirty years since the origin of these institutions in the valley of the Mississippi, and for a number of years there were but two in all this great region. The number at present exceeds a dozen, and new schools are springing up almost every year, while the increase has gone on, with nearly the same rapidity, beyond the mountains. Among unquestionable advantages that have resulted from the multiplication of these schools, there are very great evils which the profession is beginning sorely to feel. If medical education has been brought to the door of many who had not the means to go abroad in pursuit of it, the competition among institutions struggling into or for existence, the contest for students, the puffing, and the underbidding, are fast tending to deprive professional honors of all their dignity and value. Let any reader look at the following paragraphs from the Prospectus of the ***Rock** Island Medical School* lately started, and then say whether this remark is not just. We copy from the *Western Lancet.*

"That the selection of instructors has been judicious and that every member of the faculty will sustain himself before the classes may not be questioned.'" 'They have reasons (not revealed) for beleiving that a larger number of students may assemble at Rock Island than has ever composed the first class of any medical school in the Union, and that the resulting graduating class may be larger."

"No fancy sketch which the imagination can draw can compare with the beauty of the site; and its eligibility, when the Mississippi shall be bridged, can only be rivalled by its beauty. The bridging of the river at this point is so familiarly spoken of by the inhabitants as an improvement and work of art certain to take place before the elapse of many years, that the prediction may not be looked upon as an idle day-dream, that a flourishing Medical School and Hospital *may,* ere long, occupy the very same grounds on which General Taylor was ordered a few years ago to seize and erect a fort in the then wilderness and hostile Indian country. It *may not* come to pass during the presidency of General Taylor, whose policy as far as regards internal improvements, is not very lucidly revealed; neither during the presidency of General Cass, whose letter on river and harbor improvements was certainly laconic; nor yet indeed under the second term of service of the 'northern man with southern principles,' ex-president Van Buren, whose whereabouts on these small questions of river, lake or island improvements is not as well understood of late, as are his views on the great 'Wilmot Proviso question.' But nevertheless at no very remote period, under the administration of some wise president, these things *may* all come to pass on Rock Island. and medicine flourish in this panorama, par excellence, of beauty and promise, where but a few years ago, a lone military post, in the heart of the Indian country, was the solitary herald of civilization."

"The professor of the Theory and Practice has 'views on malarious fevers peculiarly his own, and which differ essentially from the doctrines of the books, and their correctness is proved from the uniform success of his practice, and that of the numerous pupils who have read medicine with him.' Happy pupils! O most excellent master! 'No student ever forgets the doctrines and precepts he receives from this master.' *Quantum sufficit.*

"The professor of Materia Medica has 'a thorough acquaintance with his subject' and moreover 'his happy arrangement disarms materia medica of much of the dryness and confusion incident to the old method of teaching still pursued in most of the schools."

In conclusion, the professors propose to receive *"obligations"* from such students as have no money.

But bad as this is, it is not the worst aspect of the case. A sorer evil is adverted to by Prof. Eve, in his introductory lecture, as having grown out of the scramble for students. He says—

"In Philadelphia there are five medical colleges, which causes a competition and contention for pupils derogatory to the profession. In vain do other colleges reject unqualified candidates, when they have only to go to Philadelphia, to be certain of a diploma in a few months, however limited their qualifications. These facts are too notorious to require scruple or delicacy in adverting to them.

"While in Philadelphia, we were informed, that a student who had been rejected by the University obtained a diploma, a fortnight after in that city. In no other city does the same corruption, do the same enormous abuses exist. It is certainly more incumbent on the physicians of Philadelphia than all others to be active and energetic in the work of reformation."

A similar statement was made to us by gentlemen in Baltimore and Philadelphia last spring, and the character of the authors leaves no room to doubt its truth. Here then, at the emporium of medical science in America, in the city of Rush, Wistar, and Physick, is an institution, chartered by the commonwealth, in which the rejected candidates of other medical schools, after a few week's study, are invested with all the honors of the doctorate. The fact ought to be proclaimed abroad, that physicians when they send their sons and pupils to Philadelphia may know what cautions to give them. By a letter just received from a physician of Mississippi who is passing the winter in Philadelphia, we are glad to see that students are fully apprised of the standing of this institution. "They look upon it," he says, "with perfect contempt. Two of the professors left it after delivering their introductories, and —— lectures on three different branches himself. He cannot get respectable physicians of the city to join him." Dr. Burden, and Dr. Thomas D. Mitchell resigned their chairs in this school at the close of the last summer session.

From the same gentleman, we learn that the Franklin Medical College has closed doors, and that the building has been sold to the Catholics for a hospital.

And so it becomes apparent, that there are limits to the capacity even of Philadelphia to support medical schools. With its teeming population and its great renown, it has not been able adequately to sustain half the number started. Its oldest schools are prosperous, and, there is every reason to believe, will continue to prosper; but the business, to use a merchant's phrase, has been overdone, and failure, and loss, as might have been foreseen, is the issue.

It were well if other cities would profit by the experience of Philadelphia. At the last session of the Legislature, of Kentucky, application was made for the privilege of establishing a second medical school in Louisville. Wisely, as we think, the application was refused, and so the evil to the city and the profession was at least postponed. If Philadelphia and New York have proved unequal to the task of maintaining more than two schools; if Kentucky has shown that she cannot support two; what can exceed the folly of attempting to build up a second one in Louisville, with less than a sixth of the population of the last of those cities.

